# Can Pharmacogenetic Variants in *TPMT*, *MTHFR* and *SLCO1B1* Genes Be Used as Potential Markers of Outcome Prediction in Systemic Sclerosis Patients?

**DOI:** 10.3390/ijms24108538

**Published:** 2023-05-10

**Authors:** Marina Jelovac, Nikola Kotur, Bojan Ristivojevic, Djordje Pavlovic, Vesna Spasovski, Nemanja Damjanov, Sonja Pavlovic, Branka Zukic

**Affiliations:** 1 Institute of Molecular Genetics and Genetic Engineering, University of Belgrade, Vojvode Stepe 444a, 11000 Belgrade, Serbia; 2 Institute of Rheumatology, 11000 Belgrade, Serbia; 3 Medical School, University of Belgrade, 11000 Belgrade, Serbia

**Keywords:** systemic sclerosis, pharmacogenetics markers, methotrexate, azathioprine, rheumatologic diseases, personalized therapy

## Abstract

Systemic sclerosis (SSc) is a rare connective tissue disorder with highest morbidity and mortality among rheumatologic diseases. Disease progression is highly heterogeneous between patients, implying a strong need for individualization of therapy. Four pharmacogenetic variants, namely *TPMT* rs1800460, *TPMT* rs1142345, *MTHFR* rs1801133 and *SLCO1B1* rs4149056 were tested for association with severe disease outcomes in 102 patients with SSc from Serbia treated either with immunosuppressants azathioprine (AZA) and methotrexate (MTX) or with other types of medications. Genotyping was performed using PCR-RFLP and direct Sanger sequencing. R software was used for statistical analysis and development of polygenic risk score (PRS) model. Association was found between *MTHFR* rs1801133 and higher risk for elevated systolic pressure in all patients except those prescribed with MTX, and higher risk for kidney insufficiency in patients prescribed with other types of drugs. In patients treated with MTX, variant *SLCO1B1* rs4149056 was protective against kidney insufficiency. For patients receiving MTX a trend was shown for having a higher PRS rank and elevated systolic pressure. Our results open a door wide for more extensive research on pharmacogenomics markers in patients with SSc. Altogether, pharmacogenomics markers could predict the outcome of patients with SSc and help in prevention of adverse drug reactions.

## 1. Introduction

Systemic sclerosis (SSc) is chronic, autoimmune disorder that affects multiple organ systems. Even though SSc is a rare disease, it contributes to populations’ morbidity and mortality more than other rheumatic diseases [1]. With a 10-year survival rate between 50 and 84% SSc represents rheumatologic disease with the highest mortality rate, with scleroderma-associated interstitial lung disease (SSc-ILD) and pulmonary hypertension (PH) being most common causes of death [2]. Clinical presentations of patients are heterogeneous, but are mainly characterised by connective tissue abnormalities, microvascular damage and production of autoantibodies. Heterogeneity exists in disease types, with diffuse SSc being the type with the worst prognosis compared to localized SSc and sine scleroderma [2]. Other than aforementioned ILD and PH, common symptoms of disease progression are digestive disorders, skin thickening, appearance of digital ulcers etc. Many of these symptoms are associated with the presence of particular genetic variants, for instance, allele C in *IL-6* gene variant -174C/G is associated with severe gastrointestinal complications [3]. What is more, frequency and mortality of patients with SSc depend on patients’ sex and ancestry. Females are more prone to developing autoimmune disorders including SSc compared to males, however, clinical presentation in males appear to be more severe [4,5]. Furthermore, it has been shown that severity of SSc symptoms could be population specific [6,7,8].

Disease progression varies among patients as well, suggesting a strong need for personalized approach. Individualization of therapy according to specific genetic signature of patient relies on pharmacogenomics principles like identification of genetic variants called pharmacogenetic variants (pharmacovariants) or pharmacogenetic markers (pharmacomarkers). These have already been incorporated in the treatment of various diseases, including pediatric acute lymphoblastic leukemia (ALL) [9,10,11,12,13,14], or autoimmune disorders like rheumatoid arthritis (RA) [15,16,17] and inflammatory bowel disease (IBD) [18,19]. Since there is no efficient symptom modifying treatment for patients with SSc, therapy is administered to keep the symptoms under control. Medicaments used in management of SSc are repurposed from other diseases where similar clinical pharmacologic effect is needed. As symptoms and progression of disease vary among patients, so does the therapy management. Treatment strategy is determined based on the disease type, extent to which organs are affected and activity of the disease [20]. Initial therapy is prescribed to manage early symptoms, followed by medications that should maintain improvement. First-line therapeutics include immunosuppressive agents, most commonly used being methotrexate (MTX), glucocorticoids (GC) such as prednisone or alkylating agents cyclophosphamides (CYC). Methotrexate is an aminopterin derivate that acts as folic acid antagonist [13]. Through a complex pathway of competitively inhibiting folate cycle enzymes, MTX leads to reduced production of components needed in the synthesis of nucleoside bases, thus inducing cell death. This way, MTX can suppress overproduction of immune cells and control excessive inflammation in patients with autoimmune diseases. Immunosuppressive medicaments are used in maintenance therapy as well, usually including mycophenolate mofetil (MMF) or thiopurine drugs, for instance azathioprine (AZA). Similarly as MTX, AZA takes part in regulation of inflammation. After administration, through series of conversion, azathioprine’s metabolites incorporate into DNA and RNA molecules and block synthesis of new purines [21]. Moreover, it was shown that AZA’s metabolites induce T cell apoptosis via deactivation of a small GTPase Rac1, that is present in T lymphocytes [22]. However, drug effectiveness differs among patients, and some could even develop serious adverse drug reactions [23,24]. Knowledge on metabolism of these drugs allowed for candidate gene and pathway-based studies [17,21,25], while genome-wide association studies (GWAS) uncovered more potentially relevant response pharmacomarkers associated to drug response [26,27,28]. To date, not much can be concluded on the long-term efficacy of these therapeutics in SSc patients specifically, let alone on the impact genetic variation of these patients can have to therapy response or outcome of the disease.

The aim of our study was to investigate the association of four pharmacogenetic variants, namely *TPMT* rs1800460, *TPMT* rs1142345, *MTHFR* rs1801133 and *SLCO1B1* rs4149056 correlated with AZA and MTX respectively, with the risk of severe disease outcome in patients with SSc, defined as pulmonary fibrosis (PF), kidney insufficiency, right ventricle systolic pressure (RVSP), hypocomplementemic urticarial vasculitis (HUV) and ratio between forced vital capacity (FVC) and diffusing capacity of carbon monoxide (DLCO). Additionally, we aimed to assess the risk for developing severe outcomes of SSc using polygenic risk score (PRS).

## 2. Results

### 2.1. Demographic and Clinical Characteristics

Group of 102 patients included in this study predominantly consisted of women (87 women and 15 men). Median age was 59.5 with interquartile range 53.25–63. Most patients (39) developed PF, while FVC/DLCO > 1,6 was the least represented of five followed outcomes. No statistical significance was found in occurrence of severe diseases outcomes between patients that received different medications. All demographic and clinical factors of patients divided in groups based on prescribed therapies are shown in Table 1.

### 2.2. Association of Genetic Variants with the Risk of Severe Disease Outcome

Patients with SSc were genotyped for variants in *TPMT*, *MTHFR* and *SLCO1B1* genes. Distributions of all variants analysed in this study corresponded to Hardy-Weinberg equilibrium. Allele frequencies corresponded to those reported for European population in 1000 Genome Project Phase 3. Genotype and allele frequencies of patients with SSc are shown in Table S2 in Supplementary Materials.

Distributions of genotypes were compared between groups of patients who received different therapies. To increase statistical power of the study, a dominant genetic model was applied, such that the group that included homozygous carriers of the variant associated with normal enzymatic function was set to be a referent group in the logistic regression model. No association was found in any of the followed groups between severe outcomes of SSc and two analysed variants in *TPMT* gene (rs1800460 and rs1142345), only a trend was noticed for variant in *TPMT* rs1800460. Patients who were treated with MTX and carried alternative allele had 2 times higher risk for developing elevated RVSP (OR = 2.11 [0.897–4.984], *p* = 0.095 after adjustment for confounding factors).

Variant allele in *MTHFR* gene was shown to increase the risk for high RVSP by around 1.5 times when testing was performed in all patients, patients on AZA therapy and patients on other types of medicaments, even after adjusting for age and sex (all patients: OR = 1.19 [1.006–1.43], *p* = 0.045; AZA: OR = 1.95 [1.18–3.25], *p* = 0.02; Other therapies: OR = 1.45 [1.11–1.89], *p* = 0.03). Furthermore, *MTHFR* rs1801133 variant was associated with decreased risk for kidney insufficiency in patients treated with different therapeutics than MTX and AZA after adjustment (OR = 0.74 [0.55–0.989], *p* = 0.049).

**Figure 1:**
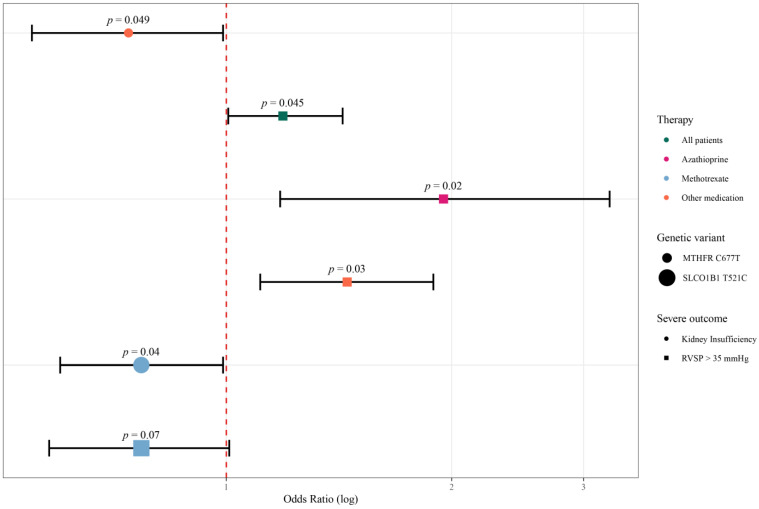
Pharmacogenetic variants associated with severe outcomes in patients with SSc represented with forest plot. Only variants that remained associated with severe outcome after adjusting for age and sex in logistic regression model are shown. Odds ratio (or risk ratio) is represented with circle or square, depending on genetic variant it represents, while 95% confidence interval is represented with whiskers. Red line illustrates the line of null effect, such that values to the right show increased risk of severe outcome, while the ones to the left depict lower risk. RVSP, right ventricle systolic pressure.

As for the variant in *SLCO1B1* gene, association of variant allele and lower risk for developing kidney insufficiency and high RVSP was observed in group of patients being treated with MTX (OR = 0.76 [0.59–0.96], *p* = 0.03 and OR = 0.74 [0.56–0.97], *p* = 0.04 respectively) and with developing PF in patients treated with AZA (OR = 0.64 [0.43–0.95], *p* = 0.04). Moreover, same variant was shown to increase the risk for developing HUV in patients who were treated with medication other than AZA and MTX (OR = 1.22 [1.05–1.41], *p* = 0.01). However, only the association of this variant and development of kidney insufficiency remained statistically significant after adjustment for age and sex (OR = 0.77 [0.60–0.99], *p* = 0.04]. All results are shown in Table S3 of Supplementary Materials. Association of analysed genetic variants and severe outcomes that proved to be statistically significant or even remained statistically significant after adjusting for confounding factors is presented in Table 2 and in Figure 1.

### 2.3. Polygenic Risk Score for Assessing Severe Disease Outcomes

To assess the risk for developing severe outcomes in patients with systemic sclerosis, polygenic risk score (PRS) was calculated (Table 3), in such a way that each variant analysed in this study was assigned points based on the prospective influence it has on enzymatic activity. GWAS [26,28,29] were searched for impact of previously described variants on protein function and β values from these studies were implemented into PRS calculation. Ranking of patients with SSc based on PRSs did not show statistically significant differences in experiencing severe symptoms of disease. However, for patients with SSc receiving MTX a trend was shown for having a higher PRS rank and elevated RVSP (*p* = 0.0838).

## 3. Discussion

Over the last decade personalized medicine has been presented as a holistic approach to improve patients’ outcome. Even with the same diagnosis, a need for optimizing therapies based on patient’s unique clinical features and genetic background was described for many diseases, including different types of cancer and autoimmune disorders (AD) [9,30,31]. Systemic sclerosis, a highly heterogeneous disease, among the most under-researched AD with high mortality rate and no efficient disease tailored therapy, represents one of the diseases whose patients would significantly benefit from introducing pharmacogenomics into daily clinical routine. Use of predictive pharmacogenomics markers to individualize the dose of the drugs would greatly improve the overall treatment outcome.

Since high heterogeneity exists both in clinical presentation and therapy response, traditional one-size-fits-all approach is highly outdated. In the recent years, many authors published extensive reviews on novel therapeutic options for patients with SSc [1,32,33]. Need for a hollistic approach is emphasized, such that patients with involvment of multiple organs are treated with a combination of therapeutics targeting each organ affected. For more personalized treatment, knowing the pharmacogenetic variants relevant for all drugs used in therapy management of SSc would be of great importance, especially when determining dosage of combined therapeutics in a way that it would not be harmful for a patient. Identification of pharmacogenetics markers prior to therapy administration could be easily used to manage the efficacy of the drugs and avoid adverse drug effects. Furthermore, presence of particular pharmacogenetic markers can influence drug efficiency or cause unwanted adverse reactions, thus affecting the disease course and outcome. Knowing them before the therapy administration could help to better stratify the patients, apply therapy protocols that fit to individual patients according to their genetic background. However, knowledge on pharmacogenetic markers as potential predictors of outcome of the disease in patients with SSc is still scarce.

Immunosuppressive drugs, such as AZA and MTX, are quite well-studied in terms of their metabolism’s dependency on individual’s genotype [34] and few variants already emerged as significantly associated with levels of these drugs in several GWA studies [26,28,29]. Research presented herein is the first study focusing on pharmacogenetics of patients with SSc from Serbia. Patients were treated either with immunosuppressants MTX and AZA or with other types of medications used in SSc management, mainly GCs and CYC. Our aim was to analyse if genetic variants in genes coding for enzymes involved in MTX and AZA metabolic pathways are associated with disease outcome in patients with SSc following MTX and AZA therapy. Since immunosuppressants are of utmost significance in SSc management [35], and MTX and AZA metabolic pathways are well-described, patients with SSc receiving immunosuppressive drugs were central to our study. Notably, we focused on association of pharmacogenetic markers to outcome of the disease rather than to adverse drug reactions, because the former is highly underestimated in SSc research. Moreover, we aimed to assess genetic risk for developing severe outcomes of SSc using available data from meta-studies and constructing PRS model.

Most patients in Serbian cohort were female (87%), as expected, since ADs, including SSc, predominantly affect women [4,5]. Predominance of female patients with SSc might be due to females being carriers of two copies of the X chromosome, which contains *IRAK1* gene associated with higher risk for developing SSc [36]. Even though the percentage of women and men in our study is a good representation of SSc patients in Serbia overall, the influence of sex on disease progression was probably strong enough to confound possible effect of genetic variants on therapy response. For example, hypocomplementemic urticarial vasculitis (HUV) was noticed in a small number of patients, exclusively women. Hence, it was impossible to associate selected genetic variants (rs4149056) to this outcome, without it being strongly influenced by sex (*p* = 0.01 before adjustment for age and sex, *p_adj_* = 0.197).

Population-wise, patients with African-American ancestry develop SSc more frequently and express more severe symptoms than Caucasians [4]. Increased prevalence of systemic sclerosis in African-Americans might be partially due to a higher frequency of HLA-DRB1*08:04 and HLA-DRB1*11:02 alleles, which are also associated with higher risk for SSc development [6]. Asian populations are considered to be the least frequently affected by this multisystemic disease [7], but are prone to developing more severe SSc types [8].

As demonstrated pharmacogenetic markers of response to initial therapy with MTX and maintenance therapy with AZA in other diseases with proven roles in action and clearance of these medicaments [21,24,25,37,38,39], variants in *TPMT, MTHFR* and *SLCO1B1* genes (rs1800460, rs1142345, rs1801133 and rs4149056 respectively) were selected for investigation in patients with SSc.

Even though all patients were genotyped for all 4 variants, every patient that was heterozygous carrier of rs1800460 was also a carrier of one rs1142345 variant in *TPMT* gene. Hence, all results were the same for these two variants, consequently only 3 variants were further assessed as pharmacogenetic markers. Haplotype TPMT*3A containing two nonsynonymous single nucleotide polymorphisms *3B and *3C (variants rs1800460 and rs1142345) is the most frequent genetic variation in *TPMT* gene, including Serbian population [34], thus finding only *3B or *3C individuals was not likely. Our conclusion that all patients with SSc were carriers of TPMT*3A instead of *3B or *3C is aligned with the fact that no severe side effects (e.g., myelosuppresion or neutropenia associated with thiopurine administration) were reported following the treatment with AZA. Carriers of alternative TPMT*3A allele have decreased TPMT enzymatic activity when thiopurine drugs like AZA are administered. This lack of TPMT activity produces toxic metabolites which cause unwanted adverse side effect on patients. For this reason *TPMT* genotyping is already recommended prior to treatment commencement. However, toxicity of mentioned drugs was not the focus of our study, but rather drug effect on individuals based on their genotype and capability of processing these medicaments. We expected that with decreased activity drugs would be less successful in disease management and patients carrying alternative alleles would be at greater risk of developing severe outcomes of SSc. Yet no significant results were noticed for variants in *TPMT* gene in our study. Only a trend was seen regarding higher risk of elevated RVSP in patients with SSc that were heterozygous carriers of TPMT*3A prescribed with MTX. This was not completely unexpected, since it was shown [40] that MTX can decrease enzymatic activity of TPMT via binding to an active enzyme site. Impaired TPMT enzyme’s function could complicate further administration of AZA. Lack of significant association of pharmacogenetic markers in *TPMT* gene in our patients with SSc could be conferred to small sample size and generally low frequency of minor variants in *TPMT* gene [41]. There were no homozygous carriers of *TPMT* variants, which leads us to conclusion that every patient had at least an intermediately functioning TPMT enzyme. To prevent adverse side effects, *TPMT* genotyping remains necessary, and *TPMT* genotype influence on thiopurine therapy effectiveness in various diseases including SSc ought to be analysed more thoroughly.

Another variant analysed in patients with SSc in this study was a missense variant C677T in *MTHFR* gene, rs1801133. As was the case with previously discussed variants, higher occurrence of severe SSc outcomes was expected in carriers of alternative *MTHFR* allele associated with decreased MTHFR enzyme activity. Our results have shown that this variant could be a significant pharmacogenetic marker associated with SSc treatment outcome. Lower occurrence of kidney insufficiency was shown for rs1801133 variant carriers in the group of patients with SSc not treated with immunosuppressive drugs. Patients in this group received GCs, and effectiveness of GCs was shown to be increased when alternative rs1801133 allele was present in children with bronchial asthma [42]. Potential protectiveness of rs1801133 variant allele in end stage renal crisis was mentioned throughout meta-analysis by Chang et al., however, results were contradictory among different populations [43]. Even though GCs were not our primary focus, this study showed that *MTHFR* rs1801133 might serve as a predictor of GCs response and should be further validated as such. Our results have indicated that patients with SSc, carriers of alternative rs1801133 allele, are at higher risk for developing elevated RVSP, which was significantly associated in all groups of patients with SSc except in the group treated with MTX (all patients: *p* = 0.045; Other: *p* = 0.03). Variant rs1801133 carriers treated with AZA were at 2 times higher risk of developing high RVSP (*p* = 0.02), which suggests that this variant could influence and disturb maintenance therapy with AZA. Interference of AZA might be due to the earlier discussed influence *MTHFR* variant has on TPMT enzymatic activity [44]. Lack of significant association of variant rs1801133 with high RVSP in patients with SSc treated with MTX could be explained again by small sample size, as could inconsistencies regarding protectiveness of rs1801133 variant allele be interpreted. General disagreement exists when it comes to the effect this rs1801133 variant has to MTX therapy effectiveness, which might be attributed to different frequencies of rs1801133 alleles across populations. It is necessary to validate association of variant rs1801133 with high RVSP in bigger cohorts of patients with SSc and in other populations treated with same therapeutics.

Surprisingly, a variant in *SLCO1B1* gene, T521C (rs4149056) was shown to be less frequent in patients with SSc treated with MTX experiencing kidney insufficiency (*p* = 0.04). OATP1B1 enzyme encoded by *SLCO1B1* gene is a transporter present at liver surface and partakes in drug clearance from the organism. The OATP1B1 enzyme is less effective when alternative *SLCO1B1* allele is present, so hepatic MTX uptake would be damaged. For this reason the drug clearence is prolonged and MTX could produce its effect for longer period of time. Nontheless, this could have toxic ramifications were the drug administered at higher dosage [45]. Furthermore, a trend was shown for patients with SSc *SLCO1B1* rs4149056 variant carriers treated with MTX to less frequently experience high RVSP (*p* = 0.04, *p_adj_* = 0.07). Additionally, in patients recieving AZA, *SLCO1B1* rs4149056 minor allele was protective against pulmonary fibrosis (*p* = 0.04), however this signal was completely lost after adjusting for confunding factors (*p_adj_* = 0.12). Even though alterantive allele protectiveness might be explained by longer drug exposure and similar results were present in several studies [46,47], those studies were focusing on different diseases and outcomes. Other study did not associate slow OATP1B1 transporters, or *SLCO1B1* variant carriers, with reduced risk for kidney and heart failure in Diabetic Kidney Diseases patients [48]. Results of our study concerning *SLCO1B1* rs4149056 are somewhat contradictory to previouse research focusing variant carriers as more prone to developing adverse drug reactions and less responsive to therapy [49,50]. Expected higher frequency of rs4149056 alternative allele was found in patients with SSc treated with other medications who experienced HUV (*p* = 0.01). However, after adjusting for age and sex, this association was lost (*p_adj_* = 0.197). As was mentioned earlier, HUV was only detected among three women. Since selected rs4149056 variant in *SLCO1B1* gene represents one of the best characterised variants in MTX pharmacogenetics, further analysis of its effect on treatment efficacy in SSc would be recommended. Despite of differing results across studies, rs4149056 variant in *SLCO1B1* gene should be included in genetic testing in tailoring drug dosage for the best outcome possible.

Finally, our last objective was to develop polygenic risk score model for followed outcomes. Genetic variants that produce defective enzymes would be expected in higher frequencies among patients who responded poorly to therapy. Only a trend was noticed for patients with a higher risk score to develop elevated RVSP when treated with MTX (*p* = 0.0838). This trend does support our expectations, but in light of previously communicated results, it requires more profound explanation. Variants in *MTHFR* and *SLCO1B1* genes (rs1801133 and rs4149056) inclined more towards protective effect against high RVSP in patients treated with MTX, while a trend of higher risk for elevated RVSP was present for variant rs1800460 in *TPMT* gene. Plausible explanation could be that variants rs1801133 and rs4149056 in *MTHFR* and *SLCO1B1* alone produce protectiveness, but when found together interfere with the effect of the drug. Combination of variants could impede drug exploitation in the system on one side, and on the other, drug clearance from the system, making carriers more exposed to weaker therapy efficacy and adverse drug reactions. Developing a clinically applicable PRS is complicated, because many factors affect diseases and therapy responses, and it is not easy to calculate how much each factor contributes to the outcome [51]. Furthermore, PRSs are often applicable solely to the sample in which they were developed, since different populations account for different frequencies and allele weights [52]. Even so, PRS represents a promising tool for further improvement of personalized medicine [53].

Our pioneer work on the pharmacogenetics markers in SSc encompassed a small sample size and high heterogeneity both in symptoms experience of patients with SSc and the outcome of the disease. High sex imbalance that occurs in this disease further complicates the study of genetic factors in patients with SSc. Nonetheless, associations of selected pharmacogenetic variants presented in our results give hope for their potential inclusion in valuable PRSs when managing SSc. Small sample size is the main limitation of this study. When analyzing association of less common variants and outcomes in stratified patient groups, lack of statistically significant association found in this study might not be reliable indicator of the absence of effect. Additional studies are necessary for validating results from our study in bigger samples and different populations. It would be favorable to include more genetic variants, especially when considering response to combined therapies. Moreover, data on adverse drug reactions would be imperative in deciding on drug dosage and combining therapies. Overall, not only for avoiding adverse drug reactions, pharmacogenomics markers could be used for prediction of the outcome in patients with SSc, allowing more personalized approach in treating such a heterogeneous disease.

## 4. Materials and Methods

### 4.1. Subjects Demographics and Clinical Characteristics

In this study 102 patients with SSc were recruited from Institute of Rheumatology, Clinical Center of Serbia in Belgrade, Serbia. Informed consent was obtained from all subjects involved in the study, in accordance with the Helsinki Declaration. The study was approved by The Institute of Rheumatology research Ethics board. Every patient had at least one visceral organ involvement, hence all subjects fulfilled EULAR 2013 classification criteria for SSc [54].

Subjects who participated in this study were grouped by prescribed medication to those who were treated with immunosuppressants AZA or MTX or with other drugs like cyclophosphamide and GCs. Several parameters of disease outcome were obtained following the treatment and were attributed as unfavourable therapy response: pulmonary fibrosis (PF), kidney insufficiency, right ventricle systolic pressure (RVSP) higher than 35 mmHg [55,56], hypocomplementemic urticarial vasculitis (HUV) and ratio between forced vital capacity (FVC) and diffusing capacity of carbon monoxide (DLCO) higher than 1.6.

### 4.2. Blood Sampling and DNA Extraction

Blood samples of 102 informed patients were collected into two 4.5 mL sodium citrate anticoagulant tubes (Vacutainer, Becton-Dickinson, Plymouth, UK). Genomic DNA extraction from whole peripheral blood was performed using QIAampDNA Blood Mini Kit (Qiagen GmbH, Hilden, Germany), and samples were stored at −20 °C until further analysis.

### 4.3. Genotyping

Patients who were included in this study were analysed for genetic variants in *TPMT* (rs rs1800460 G > A and rs1142345 A > G), *MTHFR* (rs1801133 C > T) and *SLCO1B1* (rs4149056 T > C) genes. Detection of variants in *TPMT* and *MTHFR* genes was performed by a polymerase chain reaction restriction fragment length polymorphism (PCR-RFLP) method, as previously described [57,58].

For variant detection in *SLCO1B1* gene, direct sequencing of PCR product using BigDye Terminator v3.1 Cycle Sequencing Kit (Applied Biosystems, Waltham, MA, USA) on 3130 Genetic Analyzer (Applied Biosystems, Waltham, MA, USA) was carried out, as explained by Kotur et al. [10].

### 4.4. Statistical Analysis

All statistical analyses were performed using software R v.4.2.1 (https://www.R-project.org/). To assess the performance of genotyping, Hardy-Weinberg equilibrium was examined for all detected variants using Chi-square test. Differences in demographic and clinical characteristics between groups of patients who received separate therapies were tested with Chi-square or Fisher exact test for discrete data. Impact of detected genetic variants on severity of SS disease outcome in groups with different treatments was analysed by odds ratio (OR) with 95% confidence interval (CI) using a logistic regression model, in order to control for confounding factors including age and sex. Dominant genetic model was applied for comparisons between genotypes, such that homozygous for major allele were compared to heterozygous plus homozygous for minor allele. Polygenic risk score was calculated with the function $f_{\beta }\left(x\right)=\frac{\sum_{i=1}^{n}\beta_{\dot{i}}g\left(x_{i}\right)}{\sum_{i=1}^{n}\beta i}$ where *x_i_* representes points given to subjects based on the number of alleles they carry, such that homozygous carriers of major allele, which was also allele that ensures normal enzymatic function, were given 0 points, heterozygotes were given 0.5 points and homozygous carriers of minor allele, associated with impaired protein function, were given 1 point. Furthermore, β values that represent the impact of each variant to reduced enzymatic efficiency were implemented from GWAS [26,28,29] and are shown in Table S1 in Supplementary Materials. Differences in distribution of calculated polygenic risk scores for impaired enzymatic function between patients were assessed with Mann-Whitney U test for continuous data. All tests in this study were bi-directional and differences were considered to be significant in all cases when *p <* 0.05.

## Figures and Tables

**Table 1:** Demographic and clinical characteristic of patients with SSc (n = 102) divided in groups depending on prescribed therapy.

	AZA	MTX	AZA + MTX	Other Medication
N^o^ of patients (%)	16 (15.7)	43 (42.2)	3 (2.9)	40 (39.2)
Age, median [IQR]	61 [58.75–63]	57 [49.5–61.5]	60 [51.5–62.5]	60.5 [54.5–66]
N^o^ of women (%)	14 (13.7)	37 (36.6)	3 (2.9)	33 (32.2)
PF (%)	6 (5.9)	14 (13.7)	0	19 (18.6)
Kidney insufficiency (%)	4 (3.9)	10 (9.8)	0	14 (13.7)
RVSP > 35 mmHg (%)	2 (1.9)	8 (7.8)	0	12 (11.8)
HUV (%)	7 (6.9)	12 (11.8)	0	12 (11.8)
FVC/DLCO > 1.6 (%)	0	4 (3.9)	0	2 (1.9)

Differences between groups were tested using Chi-square/Fisher exact test for discrete data. IQR, interquartile range; RVSP, right ventricle systolic pressure; HUV, hypocomplementemic urticarial vasculitis; FVC/DLCO, ratio between forced vital capacity (FVC) and diffusing capacity of carbon monoxide (DLCO); AZA, Azathioprine; MTX, Methotrexate; PF, pulmonary fibrosis.

**Table 2:** Association of the analysed genotypes with the risk of severe outcomes in patients with systemic sclerosis.

Genetic Variant (rs Number)	Disease Outcomes	Therapy	Dominant Genetic Model	*p* OR [CI 95%]	*p_adj_* OR [CI 95%]
TPMT*3A (rs1800460 and rs1142345)	RVSP > 35 mmHg	MTX	GG^R^ vs. GA + AA/ AA^R^ vs. AG + AA	0.11 2.09 [0.87–5.06]	0.095 2.11 [0.897–4.984]
*MTHFR* rs1801133	Kidney insufficiency	Other	CC^R^ vs. CT + TT	0.12 0.79 [0.59–1.05]	**0.049** **0.74 [0.55–0.989]**
*MTHFR* rs1801133	RVSP > 35 mmHg	All patients	CC^R^ vs. CT + TT	**0.03** **1.22 [1.02–1.45]**	**0.045** **1.19 [1.006–1.43]**
*MTHFR* rs1801133	RVSP > 35 mmHg	AZA	CC^R^ vs. CT + TT	**0.04** **1.69 [1.08–2.64]**	**0.02** **1.95 [1.18–3.25]**
*MTHFR* rs1801133	RVSP > 35 mmHg	Other	CC^R^ vs. CT + TT	**0.009** **1.45 [1.11–1.89]**	**0.03** **1.45 [1.11–1.89]**
*SLCO1B1* rs4149056	PF	AZA	TT^R^ vs. TC + CC	**0.04** **0.64 [0.43–0.95]**	0.12 0.66 [0.41–1.08]
*SLCO1B1* rs4149056	Kidney insufficiency	MTX	TT^R^ vs. TC + CC	**0.03** **0.76 [0.59–0.96]**	**0.04** **0.77 [0.60–0.99]**
*SLCO1B1* rs4149056	RVSP > 35 mmHg	MTX	TT^R^ vs. TC + CC	**0.04** **0.74 [0.56–0.97]**	0.07 0.77 [0.58–1.009]
*SLCO1B1* rs4149056	HUV	Other	TT^R^ vs. TC + CC	**0.01** **1.22 [1.05–1.41]**	0.197 1.11 [0.95–1.30]

Association of analysed genotypes with severe outcomes in patients with systemic sclerosis was tested using logistic regression model, where dominant genetic model was applied. Group that included homozygous carriers of allele associated with normal enzymatic functioning, which was also the more frequent allele, was set to be a referent group in logistic regression model. All genotyped patients had TPMT*3A haplotype, meaning that patients carrying *TPMT* rs1800460 together with *TPMT* rs1142345. Hence, all results were the same for these two variants. Bolded *p* values were considered significant. OR, odds ratio; CI, confidence interval; *p_adj_*, adjusted for age and sex; RVSP, right ventricle systolic pressure, HUV, hypocomplementemic urticarial vasculitis; FVC/DLCO, a ratio between forced vital capacity (FVC) and diffusing capacity of carbon monoxide (DLCO); AZA, Azathioprine; MTX, Methotrexate; PF, pulmonary fibrosis.

**Table 3:** Association of polygenic risk score in patients with SSc who received different types of therapy with experiencing severe disease outcomes.

Severe Outcome	*p* Values			
	All Patients	AZA	MTX	Other Medication
PF	0.4228	0.7585	0.6384	0.1409
Kidney insufficiency	0.1535	0.8091	0.3981	0.2699
RVSP > 35 mmHg	0.9052	0.5914	0.0838	0.1412
HUV	0.8329	NaN	0.5498	0.1144
FVC/DLCO > 1.6	0.3346	0.2713	0.3472	0.757

Differences in polygenic risk scores between groups of patients with SSc who have or have not experienced severe illness were tested using Mann-Whitney U test for continuous data. NaN, not a number, in the group of patients treated with AZA no-one developed HUV; PF, pulmonary fibrosis; RVSP, right ventricle systolic pressure; HUV, hypocomplementemic urticarial vasculitis; FVC/DLCO, ratio between forced vital capacity (FVC) and diffusing capacity of carbon monoxide (DLCO); AZA, Azathioprine; MTX, Methotrexate.

## Data Availability

The authors confirm that the data supporting the findings of this study are available within the article; further inquiries can be directed to the corresponding author.

## Supplementary Materials

The following supporting information can be downloaded at: https://www.mdpi.com/article/10.3390/ijms24108538/s1.

## Abbreviations

*TPMT*: thiopurine S-methyltransferase; *MTHFR*: methylenetetrahydrofolate reductase; *SLCO1B1*: solute carrier organic anion transporter family member 1B1; SSc: Systemic sclerosis; PCR-RFLP: polymerase chain reaction-restriction fragment length polymorphism; PRS: polygenic risk score; MTX: methotrexate; AZA: azathioprine; ILD: interstitial lung disease; PH: pulmonary hypertension; IL-6: interleukine 6; ALL: acute lymphoblastic leukemia; RA: rheumatoid arthritis; IBD: inflammatory bowel disease; GC: glucocorticoids; CYC: cyclophosphamides; MMF: mycophenolate mofetil; GWAS: genome-wide association studies; PF: pulmonary fibrosis; RVSP: right ventricle systolic pressure; DLCO: diffusing capacity of carbon monoxide; FVC: forced vital capacity; OR: odds ratio; CI: confidence interval; *p_adj_*: adjusted for age and sex; NaN: not a number; AD: autoimmune disorders.
