# Supplementary material for: Can Pharmacogenetic Variants in TPMT, MTHFR and SLCO1B1 Genes Be Used as Potential Markers of Outcome Prediction in Systemic Sclerosis Patients?
Source: Int J Mol Sci. 2023 May 10;24(10):8538. doi: 10.3390/ijms24108538 (PMC10217985; doi:10.3390/ijms24108538)
Supplement: Supplementary file 1 [file ijms-24-08538-s001.zip › ijms-2353241-supplementary.pdf]

Table S1. Parameters used in polygenic risk score analysis

Genetic variant (risk allele)	*TPMT* rs1800460 (A)	*TPMT* rs1142345 (G)	*MTHFR* rs1801133 (T)	*SLCO1B1* rs4149056 (C)
β	-3.26	-2.67	-0.05	-2.53

Risk allele is the allele associated with lower enzymatic activity. Homozygous carriers of the risk allele were valued with 1 point, heterozygotes with 0.5 points and homozygous carriers of allele associated with normal enzymatic activity were given 0 points. β values used in this study were extracted from genome wide association studies.

Table S2. Genotype and allele frequencies of systemic sclerosis patients (n = 102)

Genetic variant	Genotype	n (freq)	HW	Allele	Frequency in European population
TPMT rs1800460	GG<br>GA<br>AA	97 (0.95)<br>5 (0.05)<br>0	1	G<br>A	0.97<br>0.03
TPMT rs1142345	AA<br>AG<br>GG	97 (0.95)<br>5 (0.05)<br>0	1	A<br>G	0.97<br>0.03
MTHFR rs1801133	CC<br>CT<br>TT	46 (0.45)<br>41 (0.40)<br>15 (0.15)	0.3	C<br>T	0.63<br>0.37
SLCO1B1 rs4149056	TT<br>TC<br>CC	70 (0.69)<br>30 (0.29)<br>2 (0.02)	0.8	T<br>C	0.84<br>0.16

Allele frequencies in European population were reported in 1000Genome Project Phase 3. HW, Hardy-Weinberg equilibrium.

Table S3. Association of the analysed genotypes with the risk of severe systemic sclerosis outcomes.

Genetic variant (rs number)	Disease outcomes	Therapy	Dominant genetic model	*p*<br>OR [CI 95%]	p_{adj}<br>OR [CI 95%]
*TPMT**3A (rs1800460 and rs1142345)	PF	All patients	GG^R vs GA + AA<br>/<br>AA^R vs AG + AA	0.52<br>1.82 [0.23 – 11.59]	0.57<br>1.74 [0.21 – 11.67]
*TPMT**3A	PF	AZA	GG^R vs GA + AA<br>/<br>AA vs AG + AA	0.40<br>3.67 [0.13 – 114.48]	0.57<br>2.48 [0.08 – 8xe+1]
*TPMT**3A	PF	MTX	GG vs GA + AA<br>/<br>AA^R vs AG + AA	0.59<br>0.79 [0.34 – 1.85]	0.69<br>0.85 [0.38 – 1.89]
*TPMT**3A	PF	Other	GG^R vs GA + AA<br>/	0.18<br>1.95 [0.75 – 5.03]	0.42<br>1.56 [0.54 – 4.53]

			AA[R] vs AG + AA		
*TPMT**3A	Kidney insufficiency	All patients	GG[R] vs GA + AA / AA[R] vs AG + AA	0.99 8.01xe^{-8} [NA – 3.71xe^{+72}]	0.99 7.41xe^{-8} [NA – 2xe^{+70}]
*TPMT**3A	Kidney insufficiency	AZA	GG[R] vs GA + AA / AA[R] vs AG + AA	0.99 5.19xe^{-8} [NA – 3.89xe^{+243}]	0.99 4.34xe^{-8} [NA – INF]
*TPMT**3A	Kidney insufficiency	MTX	GG[R] vs GA + AA / AA[R] vs AG + AA	0.64 0.83 [0.38 – 1.82]	0.68 0.84 [0.38 - 1.87]
*TPMT**3A	Kidney insufficiency	Other	GG[R] vs GA + AA / AA[R] vs AG + AA	0.52 0.74 [0.29 – 1.86]	0.35 0.61 [0.21 – 1.70]
*TPMT**3A	RVSP>35mmHg	All patients	GG[R] vs GA + AA / AA[R] vs AG + AA	0.63 1.56 [0.198 – 9.912]	0.66 1.54 [0.18 – 10.84]
*TPMT**3A	RVSP>35mmHg	AZA	GG[R] vs GA + AA / AA[R] vs AG + AA	0.85 1.33 [0.04 – 38.59]	0.88 1.3 [0.04 – 3.9xe^{+1}]
*TPMT**3A	RVSP>35mmHg	MTX	GG[R] vs GA + AA / AA[R] vs AG + AA	0.11 2.09 [0.87 – 5.06]	0.095 2.11 [0.897 – 4.984]
*TPMT**3A	RVSP>35mmHg	Other	GG[R] vs GA + AA / AA[R] vs AG + AA	0.52 0.74 [0.29 – 1.86]	0.25 0.55 [0.20 – 1.51]
*TPMT**3A	HUV	All patients	GG[R] vs GA + AA / AA[R] vs AG + AA	0.99 3.56xe^{-8} [NA – 1.82xe^{+72}]	0.99 1.7xe^{-7} [NA – 9xe^{+113}]
*TPMT**3A	HUV	AZA	GG[R] vs GA + AA / AA[R] vs AG + AA	1 1	1 1
*TPMT**3A	HUV	MTX	GG[R] vs GA + AA / AA[R] vs AG + AA	0.75 0.91 [0.50 – 1.64]	0.87 0.96 [0.54 – 1.68]
*TPMT**3A	HUV	Other	GG[R] vs GA + AA / AA[R] vs AG + AA	0.82 0.95 [0.61 – 1.48]	0.18 0.74 [0.49 – 1.13]
*TPMT**3A	FVC/DLCO>1.6	All patients	GG[R] vs GA + AA / AA[R] vs AG + AA	0.32 2.54 [0.40 – 19.989]	0.24 3.44 [4xe^{-01} – 33.99]
*TPMT**3A	FVC/DLCO>1.6	AZA	GG[R] vs GA + AA / AA[R] vs AG + AA	0.69 1.80 [0.06 – 52.70]	0.39 5.3 [1.2xe^{-1} – 553.3]
*TPMT**3A	FVC/DLCO>1.6	MTX	GG[R] vs GA + AA / AA[R] vs AG + AA	0.15 1.99 [0.79 – 5.05]	0.17 1.85 [0.78 – 4.44]
*TPMT**3A	FVC/DLCO>1.6	Other	GG[R] vs GA + AA	0.299	0.55

			/<br>AA^{R} vs AG + AA	1.71 [0.63 – 4.67]	1.39 [0.48 – 4.03]

Genetic variant (rs number)	Disease outcomes	Therapy	Dominant genetic model	*p*<br>OR [CI 95%]	p_{adj}<br>OR [CI 95%]
MTHFR rs1801133	PF	All patients	CC^{R} vs CT + TT	0.25<br>1.11 [0.93 – 1.32]	0.22<br>1.11 [0.94 – 1.32]
MTHFR rs1801133	PF	AZA	CC^{R} vs CT + TT	0.42<br>1.21 [0.77 – 1.89]	0.74<br>1.096 [0.65 – 1.85]
MTHFR rs1801133	PF	MTX	CC^{R} vs CT + TT	0.31<br>1.14 [0.88 – 1.48]	0.63<br>1.06 [0.83 – 1.36]
MTHFR rs1801133	PF	Other	CC^{R} vs CT + TT	0.68<br>1.07 [0.79 – 1.45]	0.71<br>1.06 [0.77 – 1.46]
MTHFR rs1801133	Kidney insufficiency	All patients	CC^{R} vs CT + TT	0.97<br>0.99 [0.85 – 1.17]	0.89<br>0.99 [0.84 – 1.16]
MTHFR rs1801133	Kidney insufficiency	AZA	CC^{R} vs CT + TT	0.21<br>1.25 [0.89 – 1.74]	0.197<br>1.30 [0.89 – 1.91]
MTHFR rs1801133	Kidney insufficiency	MTX	CC^{R} vs CT + TT	0.24<br>1.16 [0.91 – 1.46]	0.34<br>1.13 [0.88 - 1.45]
MTHFR rs1801133	Kidney insufficiency	Other	CC^{R} vs CT + TT	0.12<br>0.79 [0.59 – 1.05]	**0.049**<br>**0.74 [0.55 – 0.989]**
MTHFR rs1801133	RVSP>35mmHg	All patients	CC^{R} vs CT + TT	**0.03**<br>**1.22 [1.02 – 1.45]**	**0.045**<br>**1.19 [1.006 – 1.43]**
MTHFR rs1801133	RVSP>35mmHg	AZA	CC^{R} vs CT + TT	**0.04**<br>**1.69 [1.08 – 2.64]**	**0.02**<br>**1.95 [1.18 – 3.25]**
MTHFR rs1801133	RVSP>35mmHg	MTX	CC^{R} vs CT + TT	0.64<br>0.94 [0.71 – 1.23]	0.37<br>0.88 [0.67 – 1.16]
MTHFR rs1801133	RVSP>35mmHg	Other	CC^{R} vs CT + TT	**0.009**<br>**1.45 [1.11 – 1.89]**	**0.03**<br>**1.45 [1.11 – 1.89]**
MTHFR rs1801133	HUV	All patients	CC^{R} vs CT + TT	0.28<br>0.95 [0.87 – 1.04]	0.34<br>0.96 [0.88 – 1.04]
MTHFR rs1801133	HUV	AZA	CC^{R} vs CT + TT	NaN	NaN
MTHFR rs1801133	HUV	MTX	CC^{R} vs CT + TT	0.81<br>0.98 [0.82 – 1.17]	0.47<br>0.94 [0.79 – 1.12]
MTHFR rs1801133	HUV	Other	CC^{R} vs CT + TT	0.13<br>0.90 [0.79 – 1.03]	0.26<br>0.93 [0.82 – 1.05]
MTHFR rs1801133	FVC/DLCO>1.6	All patients	CC^{R} vs CT + TT	0.52<br>1.06 [0.88 – 1.29]	0.77<br>1.03 [0.86 – 1.23]
MTHFR rs1801133	FVC/DLCO>1.6	AZA	CC^{R} vs CT + TT	0.72<br>0.91 [0.55 – 1.51]	0.96<br>0.98 [0.54 – 1.79]
MTHFR rs1801133	FVC/DLCO>1.6	MTX	CC^{R} vs CT + TT	0.91<br>1.02 [0.76 – 1.36]	0.86<br>1.03 [0.78 – 1.35]
MTHFR rs1801133	FVC/DLCO>1.6	Other	CC^{R} vs CT + TT	0.21<br>1.23 [0.89 – 1.67]	0.58<br>1.09 [0.798 –

					1.502]

Genetic variant (rs number)	Disease outcomes	Therapy	Dominant genetic model	*p*<br>OR [CI 95%]	p_{adj}<br>OR [CI 95%]
SLCO1B1 rs4149056	PF	All patients	TT[R] vs TC + CC	0.71<br>0.96 [0.799 – 1.165]	0.38<br>0.92 [0.76 – 1.11]
SLCO1B1 rs4149056	PF	AZA	TT[R] vs TC + CC	**0.04**<br>**0.64 [0.43 – 0.95]**	0.12<br>0.66 [0.41 – 1.08]
SLCO1B1 rs4149056	PF	MTX	TT[R] vs TC + CC	0.85<br>0.97 [0.74 – 1.28]	0.89<br>1.02 [0.79 – 1.32]
SLCO1B1 rs4149056	PF	Other	TT[R] vs TC + CC	0.26<br>1.22 [0.87 – 1.72]	0.62<br>1.11 [0.74 – 1.66]
SLCO1B1 rs4149056	Kidney insufficiency	All patients	TT[R] vs TC + CC	0.14<br>0.88 [0.74 – 1.04]	0.09<br>0.86 [0.72 – 1.02]
SLCO1B1 rs4149056	Kidney insufficiency	AZA	TT[R] vs TC + CC	0.86<br>1.03 [0.73 – 1.456]	0.85<br>1.04 [0.69 – 1.59]
SLCO1B1 rs4149056	Kidney insufficiency	MTX	TT[R] vs TC + CC	**0.03**<br>**0.76 [0.59 – 0.96]**	**0.04**<br>**0.77 [0.60 – 0.99]**
SLCO1B1 rs4149056	Kidney insufficiency	Other	TT[R] vs TC + CC	1<br>1 [0.71 – 1.40]	0.99<br>0.99 [0.67 – 1.48]
SLCO1B1 rs4149056	RVSP>35mmHg	All patients	TT[R] vs TC + CC	0.21<br>0.88 [0.73 – 1.07]	0.12<br>0.86 [0.71 – 1.04]
SLCO1B1 rs4149056	RVSP>35mmHg	AZA	TT[R] vs TC + CC	0.95<br>0.98 [0.58 – 1.66]	0.969<br>0.99 [0.51 – 1.89]
SLCO1B1 rs4149056	RVSP>35mmHg	MTX	TT[R] vs TC + CC	**0.04**<br>**0.74 [0.56 – 0.97]**	0.07<br>0.77 [0.58 – 1.009]
SLCO1B1 rs4149056	RVSP>35mmHg	Other	TT[R] vs TC + CC	1<br>1 [0.71 – 1.40]	0.96<br>0.99 [0.67 – 1.46]
SLCO1B1 rs4149056	HUV	All patients	TT[R] vs TC + CC	0.32<br>1.05 [0.95 – 1.16]	0.697<br>1.02 [0.93 – 1.12]
SLCO1B1 rs4149056	HUV	AZA	TT[R] vs TC + CC	NaN	NaN
SLCO1B1 rs4149056	HUV	MTX	TT[R] vs TC + CC	0.74<br>0.97 [0.80 – 1.17]	0.91<br>0.99 [0.82 – 1.19]
SLCO1B1 rs4149056	HUV	Other	TT[R] vs TC + CC	**0.01**<br>**1.22 [1.05 – 1.41]**	0.197<br>1.11 [0.95 – 1.30]
SLCO1B1 rs4149056	FVC/DLCO>1.6	All patients	TT[R] vs TC + CC	0.44<br>1.08 [0.88 – 1.33]	0.46<br>1.08 [0.89 – 1.31]
SLCO1B1 rs4149056	FVC/DLCO>1.6	AZA	TT[R] vs TC + CC	0.72<br>1.09 [0.66 – 1.83]	0.969<br>0.99[0.53 – 1.82]
SLCO1B1 rs4149056	FVC/DLCO>1.6	MTX	TT[R] vs TC + CC	0.33<br>1.16 [0.86 – 1.58]	0.24<br>1.19 [0.89 – 1.58]
SLCO1B1 rs4149056	FVC/DLCO>1.6	Other	TT[R] vs TC + CC	0.86<br>1.03 [0.72 – 1.49]	0.82<br>1.05 [0.70 – 1.56]

Association of analyzed genotypes with severe outcomes of systemic sclerosis was tested using logistic regression model, where dominant genetic model was applied. Group that included homozygous carriers of allele associated with normal enzymatic functioning, which was also the more frequent allele, was set to be a referent group in logistic regression model. All genotyped patients had TPMT*3A haplotype, meaning that patients carrying TPMT c.460A also carried TPMT c.719G. Hence, all results were the same for these two variants. Bolded p values were considered significant.

NaN, not a number, in the group of patients treated with Imuran no-one developed HUV; OR, odds ratio; CI, confidence interval; p_{adj}, adjusted for age and gender; PF, pulmonary fibrosis; RVSP, right ventricle systolic pressure; HUV, hypocomplementemic urticarial vasculitis; FVC/DLCO, ratio between forced vital capacity (FVC) and diffusing capacity of carbon monoxide (DLCO); AZA, Azathioprine; MTX, Methotrexate.
